# Experimental evidence of good efficacy and reduced toxicity with peptide-doxorubicin to treat gastric cancer

**DOI:** 10.18632/oncotarget.23319

**Published:** 2017-12-14

**Authors:** Jue Zhang, Jing-Ping Yuan, Qun Wang, Li-Hua Shao, Shao-Ping Liu, Raymond A. Firestone, Ya-Ping Hong, Ji-Guo Li, Yan-Chao Xin, Yan Li

**Affiliations:** ^1^ Department of Peritoneal Cancer Surgery, Beijing Shijitan Hospital, Capital Medical University, Beijing 100038, P.R. China; ^2^ Department of Gynecologic Oncology, Hubei Maternal and Child Hospital, Wuhan 430071, P.R.China; ^3^ Department of Oncology, Zhongnan Hospital of Wuhan University, Hubei Key Laboratory of Tumor Biological Behaviors and Hubei Cancer Clinical Study Center, Wuhan 430071, P.R. China; ^4^ Nanjing Meihua Pharmaceuticals Ltd., Nanjing 210009, P.R. China; ^5^ Princeton Globalsynthesis LLC, Bristol, PA 19007, USA

**Keywords:** molecular targeted therapy, peptide-doxorubicin, prodrug, gastric cancer, experimental study

## Abstract

**Background:**

To compare the efficacy and toxicity of peptide-doxorubicin (PDOX) and doxorubicin (DOX) on nude mice models of human gastric cancer.

**Results:**

Both PDOX and DOX could significantly inhibit tumor growth compared with Control (*P* < 0.05) in both subcutaneous and orthotopic models. Animal survival was much better in PDOX group than DOX group. In peripheral blood test, PDOX group had significantly higher levels of platelets than the Control (*P* < 0.05), and lymphocyte lower than Control (*P* < 0.05). There were no significant differences on liver, kidney and cardiac function parameters among three groups (*P* > 0.05). Immunohistochemistry showed that treatment groups had much higher Tunel than Control (*P* < 0.05), and PDOX had significantly lower Ki-67 than doxorubicin and Control group (*P* < 0.01). Western blotting showed that PDOX caused much higher expressions of P53, P21, Aparf-1, pro- and cleaved-caspase 3, compared with DOX.

**Conclusion:**

Compared with DOX, PDOX has increased effects but much decreased toxicity in treating animal model of gastric cancer.

**Materials and Methods:**

Animals in subcutaneous model were randomized into Control, doxorubicin, PDOX-L, PDOX-M, and PDOX-H groups. Animals in surgical orthotopic implantation model were randomized into Control, doxorubicin and, peptide-doxorubicin groups. The animals were treated, monitored and examined following a set protocol.

## INTRODUCTION

Gastric cancer (GC) is the second most frequent cause of cancer deaths worldwide [1, 2]. Chemotherapy has a beneficial effect on survival in patients with advanced GC [3, 4]. Doxorubicin (DOX) is one of the most efficacious anticancer agent to treat GC [5, 6], through redox cycling and the generation of reactive oxygen species (ROS) [7–9]. Such mechanisms of action, however, are also responsible for dose-dependent toxicities to the heart, kidney, liver and bone marrow [10], considerably limiting its clinical applications. There is an urgent need to reduce the toxicities while maintaining the treatment effects of DOX.

Cathepsin B (Cat B) is a lysosomal cysteine protease within normal cells, but is highly up-regulated in cancer cells, particularly at the cancer invasion front, where large amount of Cat B is released from the invading cancer cells to degrade the extracellular matrix, creating a favorable microenvironment for cancer cells migration and metastasis [11, 12]. High serum level of Cat B is associated with a poor prognosis in GC patients [13], more aggressive tumor behavior and higher metastatic potential [14, 15]. Therefore, Cat B could be a potential target for anti-metastasis therapies [16], but Cat B-targeted therapies remains clinically unavailable up to now [17, 18].

**Figure 1:**
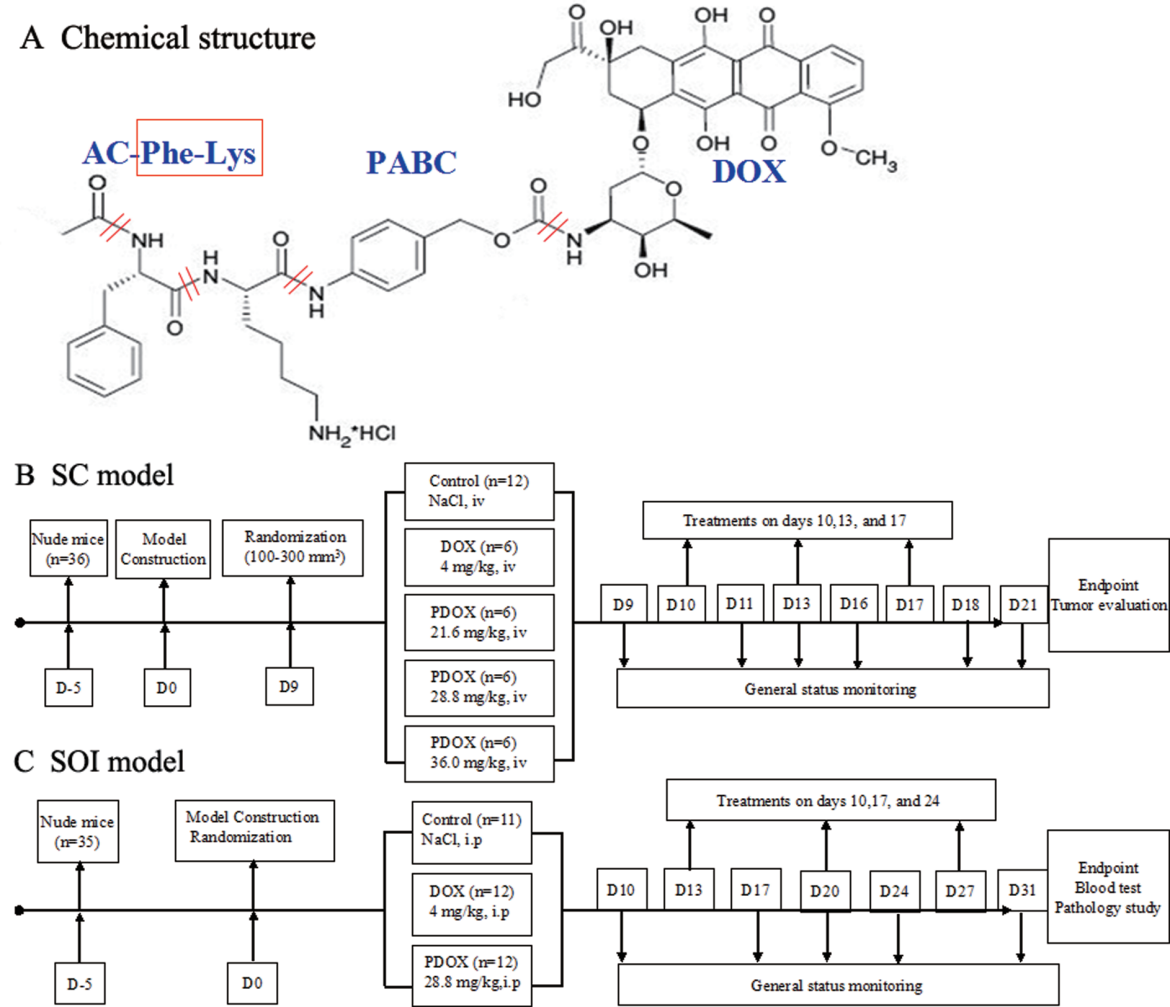
Structure and flow chart (**A**) Chemical structure of PDOX, depicting major function groups of PDOX. The design and flow chart of this study. In order to evaluate the efficacy and toxic effects of PDOX, two types of animal models were used in this study. (**B**) SC model by subcutaneous injection of BGC 823 gastric cancer cells. (**C**) SOI model of gastric cancer by surgically implanting tumor pieces of MGC 803 gastric cancer into the stomach wall of nude mice. After the models were successfully established, the animals were randomized and treated, following the description in the flow chart.

Based on the characteristics of Cat B, a smart DOX prodrug, Ac-Phe-Lys-PABC-DOX (PDOX) [19–24] has been designed, in which a Cat B-specific dipeptide (Phe-Lys) is introduced, and a self-immolative spacer para-aminobenzyloxycarbonyl (PABC) is added to increase the distance between the dipeptide and DOX, so that the dipeptide can be directly accessible to the Cat B active site (Figure 1A). In this way, the PDOX will remain stable and inactive in blood circulation [20]. When the PDOX reaches the tumor sites, the Lys-PABC bond is cleaved by Cat B, and PABC is hydrolyzed to release free DOX into the invading cancer cells. Therefore, PDOX acts as a Cat B specific drug targeting cancer invasion.

Our previous studies showed this Cat B-cleavable PDOX could enhance treatment efficacy and reduce overall toxicities on nude mice models of GC peritoneal carcinomatosis [25], liver cancer [26] and breast cancer [27]. Although the results were encouraging, one important issue remains. In previous study, the animal models of GC peritoneal carcinomatosis were constructed via direct injection of free cancer cells into the peritoneal cavity, rather than GC developed first and peritoneal carcinomatosis developed subsequently. Therefore, such animal models did not show the natural process of cancer progression from primary tumor to introperitoneal spreading.

To address such problem, in this study, we constructed the subcutaneous (SC) model and the surgical orthotopic implantation (SOI) model of human GC in which GC tissue was directly implanted into the stomach. Using such models, we further evaluated the efficacy and toxicity PDOX at escalating doses, with DOX as control. Therefore, this study was specifically designed to answer 2 questions. First, does PDOX have equal, superior or inferior effects against GC if tested on SC or SOI models? Second, what are the toxic profiles of PDOX at increased dose?

## RESULTS

### Effects on SC model

**Figure 2:**
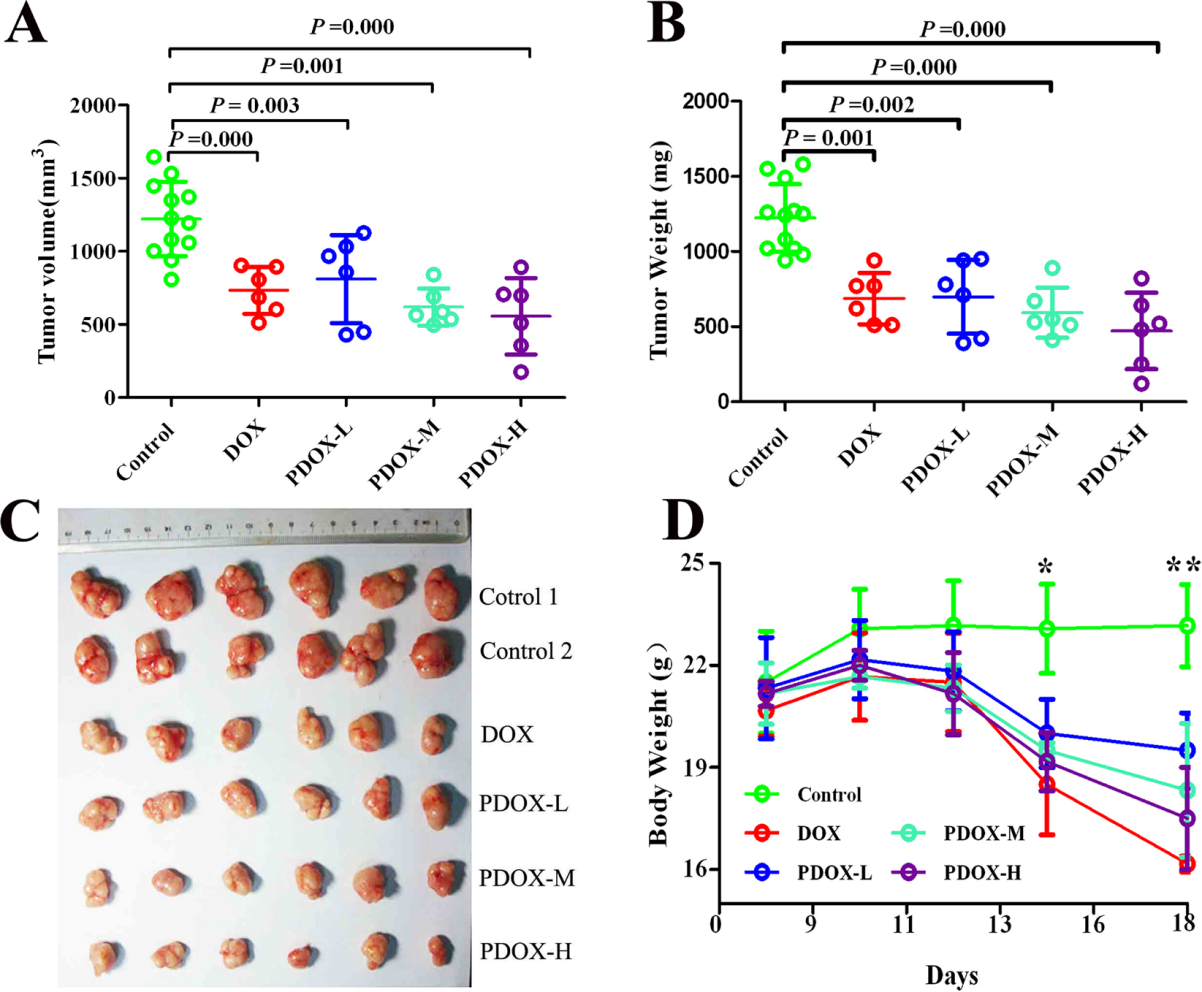
Effects of PDOX treatment on SC model of gastric cancer (**A** and **B**) Compared with control, DOX and 3 groups of PDOX treatments could significantly inhibit tumor growth. (**C**) Subcutaneous tumor sizes at the study endpoint (on day 21), showing progressively increased tumor inhibition from DOX to PDOX. (**D**) Animal body weight changes during the treatment. DOX group resulted in most significant body weight reduction compared with other groups. ^*^P < 0.05, Control group *vs.* treatment groups; ^**^P < 0.05, Control group *vs.* treatment groups; *P* < 0.05, DOX group *vs.* PDOX-L

The treatment effects of PDOX on SC model of GC were summarized in Figure 2 and Supplementary Table 1. Both the DOX group and the 3 PDOX groups showed statistically significant reductions in tumor volume and tumor weight compared with Control group. More interestingly, two observations deserve special attention. First, in terms of body weight effects, PDOX-H group (median 17.0 g, range 15.0 g–19.0 g) could result in similar body weight reduction to DOX group (median 16.0 g, range 14.0 g–19.0 g), but the much bigger tumor inhibition rate was observed in PDOX-H group (61.4%) than DOX group (43.9%). Second, in terms of tumor inhibition rate by tumor weight, PDOX-L group (42.9%) could result in similar tumor inhibition to DOX group (43.9%), but the significantly higher body weight was observed in PDOX-L group (median 18.5 g, range 15.0 g–21.0 g) than DOX group (median 16.0 g, range 14.0 g–19.0 g) (*P* < 0.05).

### Animal status in SOI model

**Figure 3:**
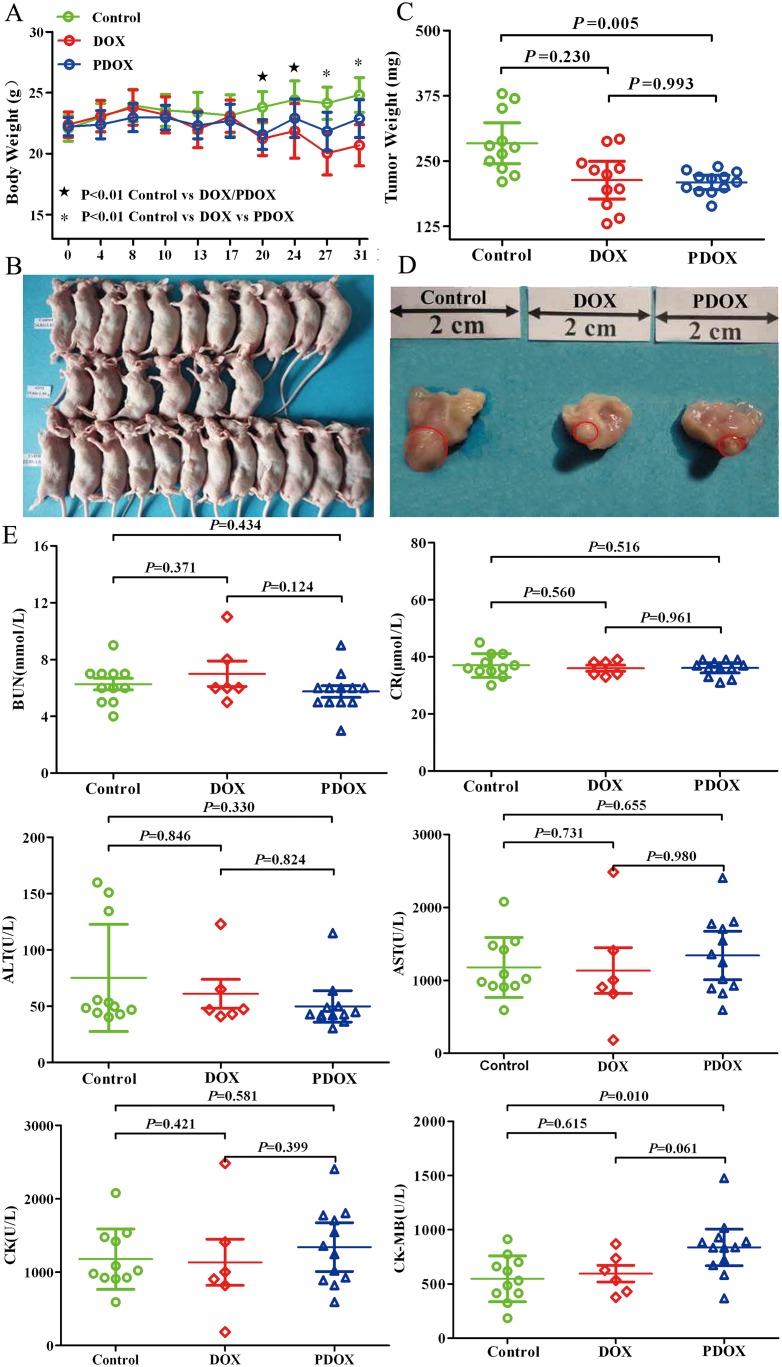
The general status of nude mice in SOI model (**A**) The DOX animals showed significant weight loss from day 20, and weight decreases became progressively greater thereafter. From day 27 on, the weight differences between the DOX and PDOX groups became statistically significantly (*P* < 0.01). (**B**) Treatment effects of DOX and PDOX on general status of nude mice. In both control (upper row) and PDOX (lower row) groups, all mice survived to the study endpoint. By contrast, only half (6/12) animals survived to the study endpoint in the DOX group (middle row). (**C**) Effects of DOX and PDOX on tumor growth in the stomach. Compared with control, PDOX resulted in 22.9% reduction (*P* = 0.005) and 18.9% reduction (*P* = 0.230) in stomach tumor weight. (**D**) representative pictures of tumor on the stomach (red circles). (**E**) Effects of different cardiac, liver and renal function parameters are shown. Only 6 samples in the DOX group because of half of mice were died. E. Toxicity of major organs in SOI model.

As shown in Figure 3, model construction was successful in all 35 animals. In control group (n = 11), all animals showed slight and steady body weight increases during the study period, and 4 (36.4%) of them had body weight increases by over 15%. In PDOX group (n = 12), 7 animals had body weight increases by 1.5% to 16.5%, and the other 5 animals had body weight decreases by 1.5% to 9.7%. By contrast, in DOX group (n = 12), only 1 animal had slight body weight increase (by 4.9%), and remaining 11 animals had body weight decreases, including 5 (41.7%) animals with body weight decreases by over 15%. Moreover, in the DOX group, 6 animals died due to severe toxicity before the study endpoint, including 1 death on day 28, 2 deaths on day 29, and 3 deaths on day 30 (Figure 3).

### Gastric tumor

As shown in Supplementary Table 2, prominent gastric tumors at the stomach body area were found in all animals, the median (range) tumor volume were 60.4 mm^3^ (12.5 mm^3^–135.7 mm^3^) in the control group, 49.9 mm^3^ (21.5 mm^3^–81.2 mm^3^) in the DOX group, 27.7 mm^3^ (15.7 mm^3^-55.4 mm^3^) in the PDOX group. Compared with control, PDOX and DOX treatments decreased the tumor volume by 54.1% and 17.4%, respectively. The PDOX group has significantly decreased tumor volume (*P* < 0.05, PDOX group *vs.* Control group), the DOX has inhibit tumor growth, but the difference was not statistically (*P* > 0.05 the DOX group *vs.* Control group) (Figure 3D, Supplementary Table 1). The median (range) tumor weights were 284.3 mg (210.5 mg–379.0 mg) in the control group, 213.5 mg (130.0 mg–292.3 mg) in the DOX group, and 209.3 mg (193.9 mg–239.4 mg) in the PDOX group (Control vs. PDOX, *P* = 0.005; Control *vs*. DOX, *P* = 0.230; DOX *vs.* PDOX, *P* = 0.993) (Figure 3C, Supplementary Table 2). Compared with control, PDOX and DOX treatments resulted in gastric tumor weight reduction 22.9% and 18.9%, respectively, which indicated that PDOX inhibited the tumor growth notably and equivalent efficacy to reduce the tumor weight compared with the DOX group.

### Toxicity studies on major organs systems

At the study endpoint, peripheral blood routine tests in all living animals were conducted to evaluate toxicities. As there were 6 animals died before the endpoint in the DOX group, we could only obtain data on the remaining 6 animals in this group (Table 1). Therefore, the data from this group as shown in Table 1 could not completely represent the real profiles of DOX adverse effects. Nevertheless, data on both control and PDOX groups were complete. Among the parameters studied, PDOX group had statistically significant higher levels of platelets, creatine kinase-myoglobin (*P <* 0.05), and had significant lower levels of lymphocyte than Control group (*P <* 0.05), but no statistically significant differences in liver and renal function parameters (*P* > 0.05, Figure 3E).

**Table 1:** Routine blood tests and biochemistry [expressed as median (range)]

Items	Control (*n* = 11)	DOX (*n* = 6)^a^	PDOX (*n* = 12)	*P* value
**Peripheral blood routine**				
RBC (T/L)	9.39 (9.17–9.84)	9.60 (8.73–12.31)	9.23 (6.65–9.75)	>0.05
WBC (×10^9^/L)	5.8 (4.6–7.9)	5.9 (4.2–20)	6.4 (4.7–7.6)	>0.05
HGB (g/L)	144 (140–153)	151.5 (145–189)	150.5 (121–157)	>0.05
PLT (×10^9^/L)	1171 (892–1666)	1291.5 (1197–1492)	1602.5 (1350–1772)^b^	<0.05
NEUT (×10^9^/L)	1.8 (1.1–1.3)	1.75 (0.7–4.2)	2.7 (1.6–3.5)	>0.05
LYM (×10^9^/L)	1.9 (1.1–3.5)^c^	1.55 (0.8–6.1)	1.25 (0.6–1.8)	=0.03
**Liver functions**				
AST (U/L)	148.1 (116.9–237.8)	136.6 (120.8–241.0)	123.4 (108.1–182.2)	>0.05
ALT (U/L)	49.8 (40.3–159.9)	47.1 (41.0–123.0)	42.9 (30.4–115.0	>0.05
**Renal functions**				
BUM (mmol/L)	6.0 (3.6–9.0)	6.2 (5.4–10.7)	5.6 (3.3–8.3)	>0.05
CR (mmol/L)	36.0 (30.0–45.0)	36.0 (33.0–39.0)	37.0(31.0–39.0)	>0.05
**Cardiac functions**				
CK (U/L)	1022.1 (592.1–2078.4)	954.05 (183.7–2485.0)	1396.9 (593.7–1804.1)	>0.05
CK-MB (U/L)	530.4 (184.8–912.7)	579.9 (375.9–868.9)	708.7 (1475.2–1016.4)^c^	=0.01
LDH (U/L)	1600 (914.6–2323.8)	1659.7 (1031.4–2027.2)	1835.3 (1448.3–2250.1)	>0.05

^a^Half of animal in DOX group died before the endpoint of the experiment, the DOX group is only 6 nude mice for blood test. The PDOX group had significant higher level than the Control group of PLT, CK-MB, significant lower of LYM (*P* < 0.05), but no statistic significant in liver and renal function (*P* > 0.05).

^b^PDOX group *vs.* Control group/DOX group; ^c^Control group *vs*. PDOX group.

### Histology study and major organs

**Figure 4:**
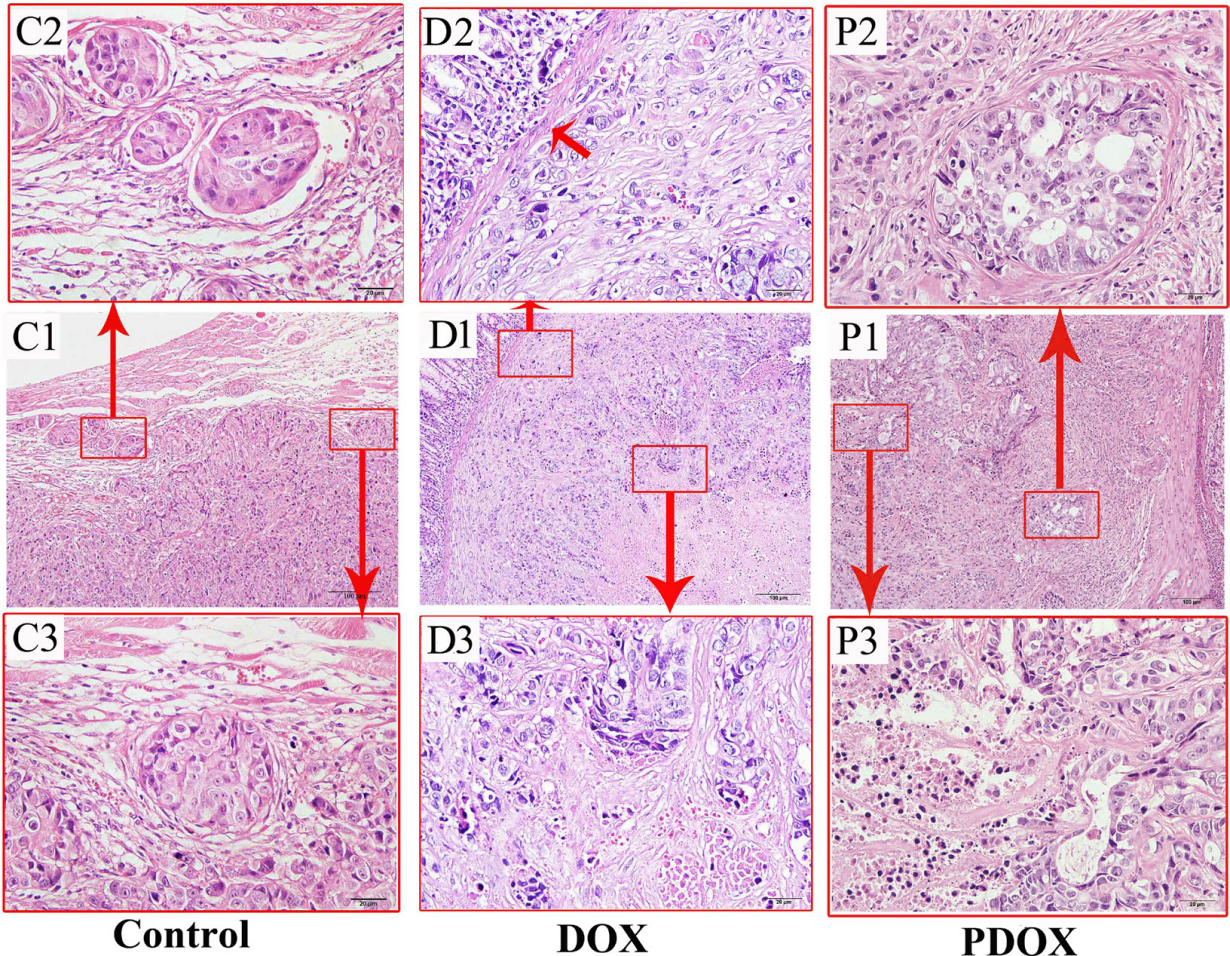
Routine histopathological features of the MGC-803 gastric cancer of different treatment groups Control group (left panel): poorly differentiated gastric carcinoma invading the whole stomach wall (C1), with prominent tumor cell emboli in the blood vessels and lymph vessels (C2), and invading tumor cell nests in the stroma (C3). DOX group (middle panel): small and scattered cancer nests invading the gastric wall (D1), with tumor cells invading the laminar proper layer of the gastric mucosa (D2), and marked tumor necrosis (D3). PDOX group (right panel): tumor nests of poorly differentiated adenocarcinoma invading the stomach wall (P1), with tumor nests surrounded by prominent fibrosis with foci necrosis of the tumor cells (P2), and extensive tumor necrosis (P3). HE staining; magnification: 10× in the middle row (scale bar = 100 μm), and 400× in the upper and lower rows (scale bar = 20 μm).

All the gastric tumors and major organs were subjected to histo-pathological studies. In control group, all gastric tumors showed large and confluent poorly differentiated tumor nests invading the whole stomach wall (Figure 4, C1), with prominent tumor thrombosis in blood vessels (9.1%) and lymphatics (72.7%) (Figure 4, C2). The polygonal tumor cells, with large oval nucleus and conspicuous nucleoli, usually form thick tumor bundles, surrounded by thin fibrous stroma (Figure 4, C3). In the DOX group, the tumor nests became much smaller and more scattered (Figure 4, D1) not penetrating the mucosal layer of the stomach (Figure 4, D2), surrounded by considerable areas of marked tumor necrosis and significant infiltration of mononuclear cells (Figure 4, D3). Tumor thrombosis in blood vessels and lymphatic were found in 54.5% (6/11) of DOX group. In PDOX group, the tumor nests were also much smaller not breaking the stomach wall (Figure 4, P1), surrounded by thick interstitial fibrosis (Figure 4, P2) and areas of complete and partial necrosis (Figure 4, P3). The tumor thrombosis in the blood vessels and lymphatics were found in 8.3% (1/12) of the PDOX (Supplementary Table 3).

As to the organ metastases, esophageal muscle metastasis was found in 1 (9.1%) mouse in Control group, but none in DOX and PDOX groups. Spleen metastases were found 2 (18.2%) mice in both Control and PDOX groups, and none in DOX group. The percentages of abdominal wall invasion were 100%, 27.3%, and 33.3%, respectively, in the Control, DOX and PDOX groups (*P* < 0.01, Control *vs.* DOX & PDOX). Compared with control, both DOX and PDOX could inhibit tumor abdominal wall metastasis by approximately 70%, and suppressed the tumor thrombosis in lymphatic by approximately 60% (Supplementary Table 3).

In terms of organ toxicities, focal myocardium mucoid degeneration was observed in 54.5% (6/11) in Control group, 54.5% (6/11) in DOX group, and 66.7% (8/12) in PDOX group (Supplementary Table 3). In addition, conspicuous spotty, focal or flaky lytic necroses in liver cells were observed in 27.3% (3/11) of DOX group, but none in the Control or PDOX.

### Immunohistological studies

**Figure 5:**
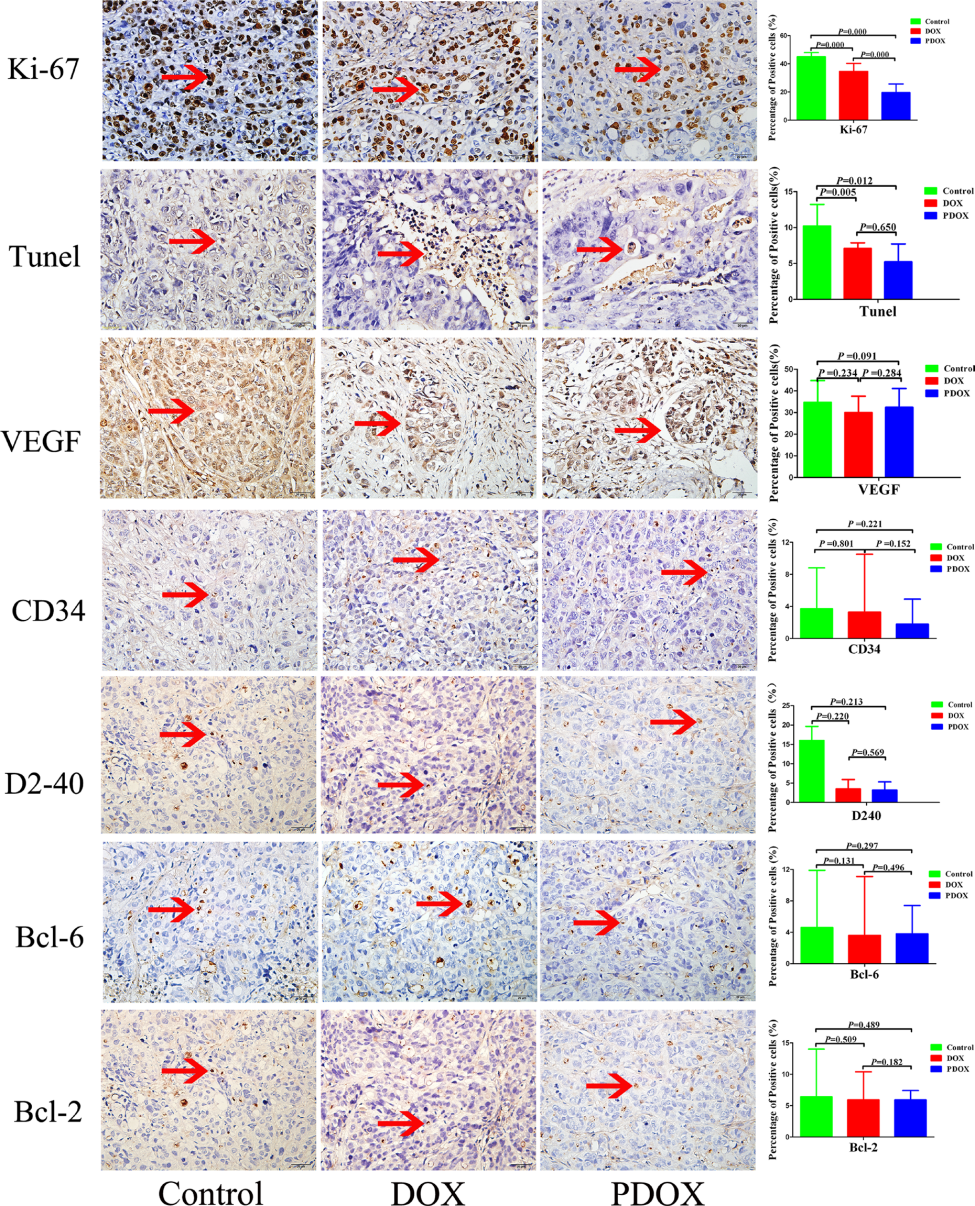
Effects of three therapies on angiogenesis, cell proliferation, and apoptosis of SOI tumor Representative pictures of blood vessels and lymphatic stained with CD34, D2-40, proliferative cells stained with Ki-67, Bcl-2, Bcl-6, and apoptotic cells stained with Tunel antibodies in Control, DOX, PDOX group. Original magnification 40×, treatment with PDOX resulted in decreased Ki-67-positive cells.

The expression of major makers of tumor proliferation, apoptosis, angiogenesis and invasion were studied with immunohistology (Figure 5). The percentage of Ki-67 in PDOX group was 2.4-fold and 1.8-fold lower than the Control and DOX groups, respectively (*P* < 0.05). Tunel was increased significantly in PDOX and DOX groups, compared with Control group (*P* < 0.05). There were no statistical differences in other parameters.

### Western blotting

As shown in Supplementary Figure 1A, both PDOX and DOX group caused up-regulation P53/P21 related apoptosis pathways. Compared with DOX, however, PDOX caused much higher expressions of P53 and caspase 3, suggesting that PDOX may have different anti-tumor mechanisms of action.

## DISCUSSION

Using both SC and SOI models of GC, this study has demonstrated that PDOX could produce increased tumor inhibition with decreased side effects, compared with DOX, the traditional cytotoxic drug in GC chemotherapy.

Several features of this study could help appreciate the PDOX from more clinically-relevant perspectives. First, this study explored two drug delivery routes, i.v and i.p, the two most common drug delivery routes to treat GC in clinical practice. Both injections could produce significant treatment effects, and more importantly no severe injection-related side effects were observed. This suggests that the PDOX could be administered via either i.v or i.p injections. Second, this study explored 3 escalating dosage scales. In the SC model, the PDOX dosages were 3×, 4×, and 5× folds of DOX in terms of equal molar dosage, and the animals could tolerate well such doses. Even at the highest dosage studied in this experiment, the animal body weight was still slightly better than the DOX group. This suggests that PDOX indeed has much overall toxicity than DOX. Moreover, when the PDOX dosage was 5× folds of DOX, the subcutaneous tumor inhibition could be enhanced by nearly 20%, while animals were still in better general conditions. Such result does support our hypothesis that we could improve efficacy by increasing the PDOX dosage. In the SOI model, the PDOX was 4 folds of DOX, and the animals could well tolerate such dosage, and did not show obvious overall toxic effects. By contrast, 6 animals in the DOX group died before the study endpoint, due to severe toxicities. All these results indicate that PDOX does have better efficacy with less toxicity. This experiment has extra significance because it involves orthotopically placed tumor, not often used because of the surgery required but more challenging to the antitumor drug, for only these tumors are growing in a favorable environment. Therefore, these tumors are stronger than SC ones and more difficult to kill.

There are other DOX-based prodrugs under development [30], such as DOXO-EMCH [28, 31], which is a macromolecule agent with superior effect over free DOX in several tumor models and is under clinical development. PK1 (FCE28068) [19] consisting doxorubicin linked to copolymers based on N-(2-hydroxypropyl) methacrylamide via a tetrapeptide spacer designed for cleavage by lysosomal cathepsins, has entered phase II/III trials. PK2 [29] (FCE28069) is designed to target the asialoglycoprotein receptor (ASGPR) which is selectively expressed in hepatocytes and hepatoma cell lines. Preclinical studies have indicated that PK2 displays 5-fold reduction in cardiotoxicity as opposed to free DOX following the i.p. and i.v. routes in various tumor models [28]. Compared with those agents, PDOX also showed better efficacy and better tolerance.

To elucidate the molecular mechanisms of PDOX, we studied both cell-cycle regulatory and cell apoptotic proteins. Immunohistology showed significantly decreased Ki-67 in PDOX group, providing convincing evidence the PDOX indeed has better anti-proliferation effects than DOX, and significantly increased Tunel in PDOX and DOX, meanwhile the Western blotting results do show more prominent up-regulation of P53 and cleaved caspase 3 in PDOX than DOX. Therefore, these results imply that PDOX could cause significant tumor apoptosis, if the treatment duration is longer. Both lines of evidence from IHC and Western blotting do suggest that PDOX exerts its anti-tumor effects at least via two mechanisms of action: direct inhibition of tumor proliferation which could produce short-term and immediate antitumor effects, and activation of P53/caspase 3-mediated apoptosis which could produce long-term and durable antitumor effects (Supplementary Figure 1B).

Of particular note are the dramatic differences in toxicity profiles between PDOX and DOX. Both SC and SOI models showed much reduced toxicities in PDOX *vs.* DOX, even though the dosage of the former was 3 to 5 times higher than the latter. The body weight changes do provide convincing evidence for the reduced overall side effects of PDOX *vs.* DOX. More evident differences were also observed in peripheral blood and biochemical studies. In the SOI model, the PDOX group had significantly higher levels of platelet and CK-MB and lower level of lymphocyte than the control group, but no statistically significant differences in liver and renal function parameters. These results may suggest: (1) PDOX itself may have some cardiac toxic effects at the dosage level 4 times higher than DOX, although our previous study found no cardiac toxicity of PDOX at the dosage level 2 times higher than DOX; or (2) when PDOX used at the dosage level 4 times higher than DOX, more DOX was released at the tumor site, and some released DOX did not enter into tumor cells, instead the released DOX was drifted away into the blood circulation, reaching the heart to cause cardiac damage. Another possibility is that we might have overwhelmed the Cat B on the cancer cells with too much drug at one time, allowing the drug itself to drift away before it gets chance to react with the Cat B. Perhaps we could reduce the amount that drifts away by using smaller infusions over a longer period of time. Future studies are warranted to investigate this possibility.

To properly understand the information from Table 1, a summary of major blood toxicities, several special considerations deserve attention. From the table itself, it could be observed that the DOX group seemingly had no significant difference compared to the control group, in terms of routine blood tests, cardiac, kidney and liver functions. However, there were only 6 animals available for these studies, and the other 6 animals died due to overt toxicities before the study endpoint. Therefore, the results are biased, and do not mean that DOX does not have toxicities; rather the results only suggest that some animals are particularly susceptible to DOX toxicity while others are not.

In conclusion, the present study has produced new evidence that PDOX is a promising Cat B targeting antitumor drug with similar efficacy and much reduced toxicities compared with DOX. This could lead to considerably increased treatment compliance and better clinical benefits to patients, if the agent could be translated in to clinical application.

## MATERIALS AND METHODS

### Agents and cells

PDOX was synthesized (by Hong YP) according to the previously reported chemical process [20–22]. The molecular weight of PDOX hydrochloride is 1046.50. In terms of equivalent mole content, 1.8 mg PDOX hydrochloride is equivalent to 1.0 mg DOX hydrochloride (molecular weight 579.99). Other agents were obtained commercially, including Doxorubicin Hydrochloride for Injection (DOX) (Pfizer Pharmaceuticals Co., Ltd at Wuxi, China), RPMI-1640 medium (HyClone, NZ, USA) and standard newborn bovine serum (ZhengZhou Ben BioTech Co., Ltd, ZhengZhou, China) for cell culture, propidine iodide (PI) agents kit (Beckman coulter, CA, USA) for flow cytometric analysis, rabbit anti-Cathepsin B polyclonal antibody (Lot No. 3190-100, BioVision, CA, USA), and peroxidase-conjugated affinipure goat anti-rabbit IgG (H+L) (Lot No. 88813, Jackson Immuno Research, PA, USA) for immunohistochemical study. The poorly-differentiated human gastric adenocarcinoma cell lines MGC-803 and BGC-823 were cultured in RPMI-1640 medium supplemented with 10% standard newborn bovine serum in the 5% CO_2_, saturated humidity, 37°C incubator (Shel Lab, OR, USA).

### Construction of GC animal models

### Animals

Male BABL/C nude mice, 6 to 8 weeks old, were purchased from Beijing HFK Bio-Technology Co. Ltd [animal quality certificate No. SCXK (Jing) 2009-0004], and maintained under specific pathogen-free conditions in an Animal Biosafety Level 3 Laboratory at the Animal Experimental Center of Wuhan University. The protocols were approved by the Animal Care Committee of Wuhan University, and the experiments were conducted in accordance with the Guidelines for the welfare and use of animals in cancer research [32].

### Rationale for the study design and key considerations

For preclinical animal studies on the efficacy and toxicities of potential new drugs against GC, several practical considerations are necessary in order to obtain convincing experimental evidence that could be translated into clinical settings. First, different GC cell lines should be used to test the potential efficacy coverage of the new agent. Second, different routes of drug delivery should be tried to test the possible way for clinical use. Third, dosage escalations should be tried to explore the optimal dosage range of the new agent.

As our previous study [25] has already tested the new agent on one GC cell line (SGC 7901, human GC adenocarcinoma cell line) in one clinical scenario (peritoneal carcinomatosis from GC) via one treatment route (intraperitoneal injection) at one dosage (7.2 mg/kg, 2 times the dosage of DOX in terms of equal mole weight), this study used other two human GC adenocarcinoma cell lines (MGC 803 and BGC 823) in two different clinical scenarios (subcutaneous tumor [SC model] and surgical orthotopic implantation gastric tumor [SOI model]), and via two clinically relevant drug delivery routes (intravenous injection and intraperitoneal injection) at a wider drug escalation scales (3, 4 and 5 times the dosage of DOX in terms of equal mole weight). Thus, such a study could cover currently practiced clinical settings. These SC and SOI models were detailed below (Figure 1B, 1C).

### SC model

BGC-823 cells were collected at the exponential growth phase. SC model was constructed by SC injecting BGC-823 cells (2 × 10^6^/per mice) into the right flank region of 36 mice on day 0. The mice were randomized into Control group (normal saline 10.0 mL/kg, n = 12), DOX group (4.0 mg/kg, n = 6), PDOX-Low group (21.6 mg/kg, n = 6), PDOX-Middle group (28.8 mg/kg, n = 6), and PDOX-High group (36.0 mg/kg, n = 6). The designed treatments via tail vein injections were started when the SC tumors reached 100-300 mm^3^. The treatment dosage was 0.4 mL/20 g body weight, tail vein injection once every three days, 3 injections for each animal, and terminated on day 21, as designed. Tumor volume were measured by the following formula: *TV* = (length × [width]^2^)/2; the inhibition $\text{rate}\left(\%\right)=1-\frac{T\text{weight}}{C\text{weight}}\times 100$, where Tweight standing for the tumor weight of treatment groups, Cweight for tumor weight of control group.

### SOI model

MGC-803 cells (5 × 10^6^/0.2 ml) were injected subcutaneously into 2 mice. After 21 days, the subcutaneous tumor reached 0.8 to 1.0 cm in diameter. After mice anaesthesia with 1.0% pentobarbital sodium solution, the tumors were removed aseptically, and fresh tumor tissues were scissor minced into pieces about 1.0 to 2.0 mm in diameter and stored in sterilized ice-cold phosphate buffered saline for SOI model construction as detailed below.

A total of 35 nude mice were used to construct SOI model. After mice anaesthesia with 1.0% pentobarbital sodium solution (20.0 mg/kg), an incision was made through left upper abdominal pararectal line and peritoneum. The stomach wall was carefully exposed, and a part of the serosal layer of the stomach, about 3.0 mm in diameter, in the middle of the great curvature of the stomach was cut open using scissors. A tumor piece was then fixed on the wound site of the serosal surface with 7-0 Dexon transmural suture. The stomach was then returned to the peritoneal cavity, and the abdominal wall and skin were closed with 4-0 Dexon sutures. After surgery, the animals were randomized into Control (n = 11), DOX (n = 12) and PDOX (n = 12) groups, and cared for according to standard protocols for 10 days when the designated treatments were conducted as detailed below.

As depicted in Figure 2C, on day 10 after SOI model construction, the mice received their designated treatments for Control group (normal saline 10.0 mL/kg, i.p, n = 11), DOX group (DOX 4.0 mg/kg, i.p, n = 12), and PDOX group (PDOX 28.8 mg/kg, i.p, n = 12). Compared with our previous study [25], in which single dosages of DOX and PDOX were 2.0 mg/kg and 7.2 mg/kg, respectively, this study increased the single dosage of DOX by 1-fold, and PDOX by 4-fold. Treatment was conducted on D10, D17, and D24, respectively. Therefore, the total dose of DOX in this study was 12.0 mg/kg, less than 16.0 mg/kg in our previous study; but the total dose of PDOX in this study was 86.4 mg/kg, higher than 57.6 mg/kg in our previous study [25]. The animals were daily monitored and body weight recorded twice a week.

On day 31 all animals were euthanized and blood was obtained for routine examinations and biochemistry study. At autopsy, the whole abdominal cavity was investigated to record tumor formation on the stomach, and formation of peritoneal carcinomatosis, including the number, size and weight of the implanted nodules, and the characteristics of ascites. The heart, lungs, liver, spleen, stomach, and intestines were obtained for routine pathologic study.

### Immunohistochemical study

To investigate the mechanisms of action, we performed immunohistochemical studies on tumor tissues from 3 groups, following our previously developed procedures [30]. The parameters included, tumor cell apoptosis markers Tunel, bcl-2/6 (Maxim- 0598, Bio Co, CHN, working solution), tumor cell proliferation marker Ki-67 (MAB-0129, Maxim-Bio Co, CHN, working solution), tumor angiogenesis CD34 (BA0532, WuHan Boster Bio-Engineering Co, CHN, 1:100), VEGF (RB-9031, Maxim-Bio Co, CHN, working solution), and tumor lymphatic marker D2-40 (AM0103, Ascend Biotechnology Co, CHN, working solution). The results determination and scoring were performed according to the method described by [33], and presented as the median and range from three animals per group.

### Toxicities study

On D31, blood was collected for biochemical study, including ALT, AST, BUN, Cr, CK, CK-MB, and LDH by Aeroset Clinical Chemistry Analyzer (Abbott Laboratories, IL, USA). At autopsy, major organs including the heart, liver, kidneys, spleen and lungs were examined for any toxic changes. Any organs involved by the tumor and the tumor nodules were formalin-fixed, for histopathological study after H&E staining.

### Western blotting analysis

To determine the changes in indicated proteins, the tumor from three groups mice were homogenized in lysis buffer, as described [30]. We immunoblotted with rabbit anti-human P53, P21, Bcl-2, Bax, Aparf-1, Caspase 3, Cleaved caspase 3, β-actin antibody (dilution 1:1,000, both from Proteintech group, USA) for 2 h. Then incubated with a peroxidase-conjugated sheep anti-rabbit IgG (dilution 1:10,000; Santa Cruz Biotechnology, Inc., Santa Cruz, CA, USA) for 1 h and blots were visualized with a chemiluminescent detection system.

### Statistical analysis

The data were analyzed on SPSS 17.0 (SPSS Inc., IL, USA). The differences in body weight and blood routine among different groups were tested using ANOVA at each time point, and the differences between every two groups were analyzed using LSD test. Because of the small sample size, blood biochemistry analysis could not fit a normal distribution of continuous data, they were given as median and range; so the two-sided non-parametric Krusal-Wallis *H*-test was used to analyze the differences among the three groups, and Mann-Whitney *U*-test was used to analyze the difference between every two groups. *P <* 0.05 was considered as statistically significant.

## SUPPLEMENTARY MATERIALS FIGURE AND TABLES



## Abbreviations

GC: Gastric cancer
SC: Subcutaneous
SOI: Surgical orthotopic implantation
ROS: Reactive oxygen species
Cat B: Cathepsin B
DOX: Doxorubicin
PDOX: Ac-Phe-Lys-PABC-DOX
PABC: Para-aminobenzyloxycarboonyl
PDOX-L: PDOX-Low dosage
PDOX-M: PDOX-Middle dosage
PDOX-H: PDOX-High dosage
iv: intravenous injection
ip: intrapertitoneal injection
SPF: Specific pathogen-free
PBS: Phosphate buffered saline
HE: hematoxylin and eosin staining
WBC: white blood cell
NEUT: neutrophil
RBC: red blood cell
HGB: hemoglobin
PLT: platelet
LYM: lymphocyte
ALT: Alanine aminotransferase
AST: Aspartate aminotransferase
BUN: Blood urea nitrogen
CR: Creatinine
CK: Creatine kinase
CK-MB: Creatine kinase-MB
LDH: Lactate dehydrogenase
H&E: hematoxylin and eosin
VEGF: Vascular endothelial growth factor receptor
AMPK: adenosine monophosphate-activated protein kinase
BW: body weight
RTV: relative tumor volume
T/C: tumor proliferation rate.
