# Supplementary material for: Experimental evidence of good efficacy and reduced toxicity with peptide-doxorubicin to treat gastric cancer
Source: Oncotarget. 2017 Dec 14;9(2):1957–68. doi: 10.18632/oncotarget.23319 (PMC5788612; doi:10.18632/oncotarget.23319)
Supplement: Supplementary file 1 [file oncotarget-09-1957-s001.pdf]

Experimental evidence of good efficacy and reduced toxicity with peptide–doxorubicin to treat gastric cancer

SUPPLEMENTARY MATERIALS

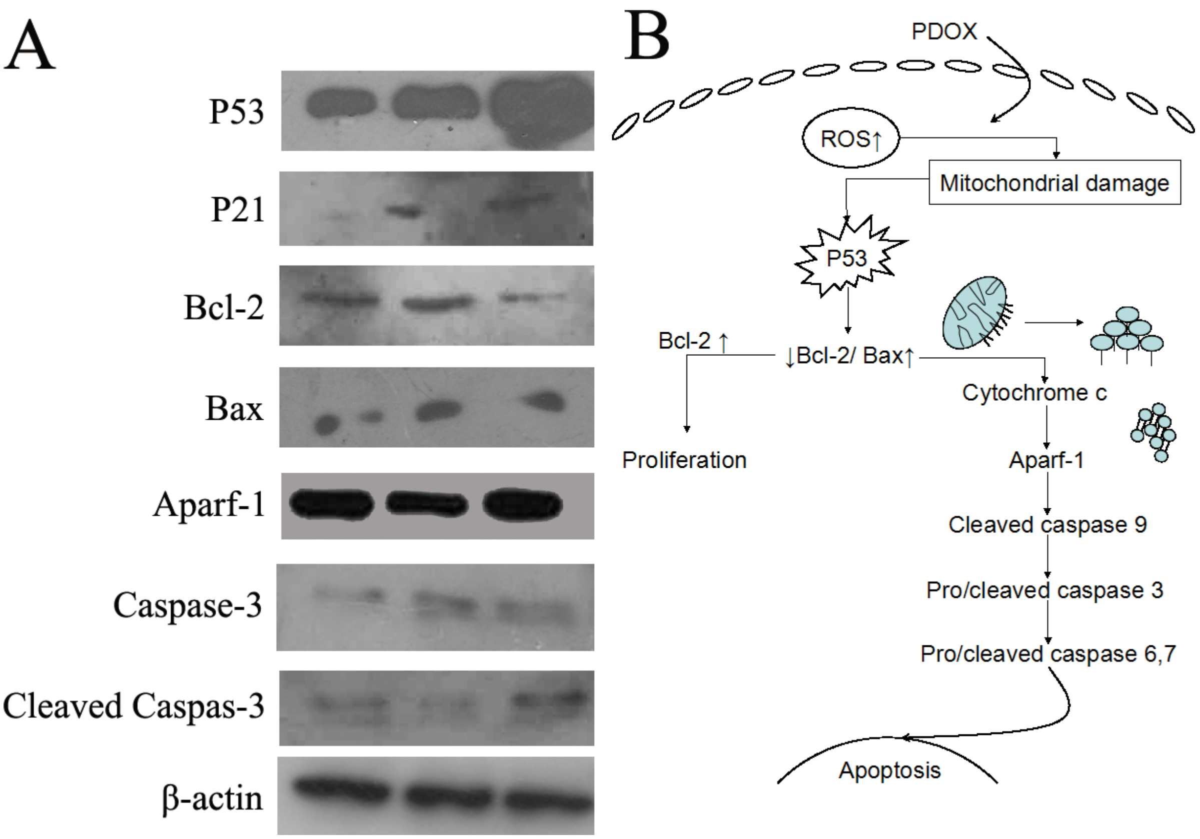

Supplementary Figure 1: Mechanism study. (**A**) Effects of PDOX and DOX on the expression of cell apoptosis regulators against SOI model. Compared with DOX, PDOX up-regulated the expressions of P53, P21, Aparf-1, pro- and cleaved- caspase 3, indicating that PDOX may have different anti-tumor mechanisms of action. (**B**) The apoptotic pathway pertinent to PDOX. The PDOX administration caused the formation of free radicals (ROS), which activate the upstream signal necessary for adenosine monophosphate-activated protein kinase (AMPK), leading to the phosphorylation of the tumor suppressor P53 can also be activated if the mitochondrial damaged as a result of PDOX administration. Up-regulate the expression of P53, P21, which the presages the outset of apoptosis. The activation of p53 triggers the migration of Bax from the cytosol to the mitochondrial surface where it forms the classic Bcl-2/Bax toggle switch complex. Bcl-2 family of proteins regulates apoptosis in mammalian cells, which acts upstream of mitochondrial caspase activation to prevent apoptosis. If the ratio of Bcl-2/Bax shifts toward Bcl-2, the apoptosis is avoided. However, if the ratio tilts towards Bax, the apoptosis pathway continues leading to cytochrome c release. The released cytochrome C binds with Aparf-1 to form a proeteasome, which leads to the sequential cleavage and activation of caspase 9 and caspase 3. Once caspase 3 is cleaved and activated, the pathway hits the point of no return, resulting in the downstream activation of caspase-6 and caspase-7 and ultimately to full-blown apoptosis.

Supplementary Table 1: The efficacy of PDOX against SC model

Items	Control group	DOX group	PDOX-L group	PDOX-M group	PDOX-H group	*P* value
**Total No**	12	6	6	6	6	
**Drug dosage**	0	1×DOX	3×DOX	4×DOX	5×DOX	
**Animal death**	0	6	1	3	4	**>0.05**
**Final BW (g)**	23.0 (21.0–25.0)	16.0 (14.0–19.0)*	18.5 (15.0–21.0)*,**	18.5 (15.0–21.0)*	17.0 (15.0–19.0)*	**<0.05**
**Tumor volume (mm^3)**	1211.1 (807.5–1645.0)	745.1 (510.3–904.0)*	912.9 (429.5–1125.1)*	575.5 (495.6–841.5)*	603.0 (173.5–891.3)*	**<0.05**
**Inhibition rate by tumor volume (%)**	0	38.5%*	24.6%*	52.5%*	50.2%*	**<0.05**
**Tumor weight (mg)**	1245.0 (940.0–1580.0)	695.0 (510.0–940.0)*	825.0 (390.0–950.0)*	460.0 (550.0–670.0)*	500.0 (120.0–820.0)*	**<0.05**
**Inhibition rate by tumor weight (%)**	0	43.9%*	42.9%*	51.5%*	61.4%*	**>0.05**

The tumor volume, BW, tumor weight and RTV are expressed as ± S; Tumor inhibition rate expressed as percentage.
*Compared with the Control group, $P < 0.05$.
*Compared with the DOX group, $P < 0.05$.
BW: body weight, RTV: relative tumor volume.

Supplementary Table 2: Effects of PDOX treatment on orthotopic tumor growth in animal model of gastric cancer

Items	Control group	DOX group	PDOX group	*P* value
**Total No**	11	12	12	–
**Drug dosage**	0	1×DOX	4× DOX	–
**Animal death**	0	6	0	**>0.05**
**Final BW (g)**	24.6 (23.2–26.9)*	21.5 (17.7–22.1)	21.7 (23.5–25.7)	**<0.05**
**Tumor volume (mm3)**	60.4 (12.5–135.7)*	49.9 (21.5–81.19)	27.7 (15.7–55.43)	**<0.05**
**Inhibition rate by tumor volume (%)**	0	17.4%*	54.1%**	**<0.05**
**Tumor weight (mg)**	276.5 (210.5–378.0)	224.2 (130.0–292.3)*	213.1 (163.9–239.4)*	**<0.05**
**Inhibition rate by tumor weight (%)**	0*	18.9%	22.9%	**<0.05**

Effects of PDOX treatment on orthotopic tumor growth in animal model of gastric cancer. The tumor volume, body weight and tumor weight were expressed as median (range).
*Control *vs.* DOX/PDOX, $P < 0.05$.
*DOX *vs.*PDOX, $P < 0.05$.

Supplementary Table 3: Metastasis of major organs and toxicities to the heart and liver in SOI model

**Items**	**Control (*n* = 11)**	**DOX (*n* = 11)**	**PDOX (*n* = 12)**
Tumor	11 (100%)	11 (100%)	11 (100%)
Tumor emboli in the lymphatic	8 (72.7%)	6 (54.5%)	1 (8.3%)**
Tumor emboli in the blood vessels	1 (9.1%)	1 (9.1%)	0
Abdomen wall spread	11 (100%)*	3 (27.3%)	4 (33.3%)
Spleen metastases	2 (18.2%)	0	2 (18.2%)
Esophageal muscle metastasis	1(9.1%)	0	0
Myocardium degeneration	6 (54.5%)	6 (54.5%)	8 (66.7%)
Necrosis of liver cells	0	3 (27.3%)	0

The metastasis of major organs in mices. Compared with control, both DOX and PDOX could inhibit tumor abdominal wall metastasis by approximately 70%, and suppressed the tumor emboli in lymphatic by approximately 60%.

*$P < 0.01$, Control *vs.* DOX /PDOX;

**$P < 0.05$, PDOX *vs*. Control/DOX.
